# PPI adverse drugs reactions: a retrospective study

**DOI:** 10.1186/s12948-019-0104-4

**Published:** 2019-01-18

**Authors:** Marco Casciaro, Michele Navarra, Giuseppina Inferrera, Marta Liotta, Sebastiano Gangemi, Paola Lucia Minciullo

**Affiliations:** 1 School and Unit of Allergy and Clinical Immunology, Department of Clinical and Experimental Medicine, University of Messina, Messina, Italy; 2 Department of Chemical, Biological, Pharmaceutical and Environmental Sciences, University of Messina, Messina, Italy; 3 University Hospital “G. Martino”, University of Messina, Messina, Italy

**Keywords:** PPI, Proton pump inhibitors, Adverse reaction, Hypersensitivity, Allergy

## Abstract

Proton pump inhibitors (PPIs) are drugs capable of blocking the gastric pump H,K-ATPase in order to inhibit gastric acid secretion. Omeprazole, lansoprazole, pantoprazole, rabeprazole and esomeprazole belong to PPIs category. Although PPIs have a good safety profile, allergic reactions to these molecules can occur. The real rate of hypersensitive reactions to PPIs is unknown. The aim of this retrospective study is to evaluate the rate of hypersensitive reactions to PPIs in patients admitted to our Unit between 2008 and 2013 with a history of drug hypersensitivity. From a database of 1229 patients (921 women, 308 men) with adverse drug reaction we extrapolated the data about PPI reactions. Twelve patients (10 female, 2 men) had a positive history for hypersensitive reaction to PPI. Pantoprazole was the most frequently PPI involved. Based on patient personal history in some cases we performed an oral challenge test for an alternative anti-acid drug and none of them had adverse reactions. According to our experience and according to the literature and pharmacovigilance reports, ADR caused by PPIs are ever increasing. Adverse reactions to these drugs are still under-reported; however, considering the frequency of their prescription worldwide, the risk of severe allergic events is low. Further studies are needed to provide clearer data on the real incidence and prevalence about this matter. This should be useful to help physician in choosing the molecule to prescribe and, in case of hypersensitivity, the alternative molecule to test, also considering the possible cross-reactivity.

## Introduction

Proton pump inhibitors (PPIs) are drugs capable of blocking the gastric pump H,K-ATPase in order to inhibit gastric acid secretion [1]. They are used to treat many diseases related to an altered gastrointestinal pH, like peptic ulcers, gastroesophageal reflux disease (GERD), Barrett’s esophagus [2]. Omeprazole, lansoprazole, pantoprazole, rabeprazole and esomeprazole belong to PPI category. They undergo a hepatic metabolism and they have no direct toxicity [3]. Although their good safety profile [4], allergic reactions to PPIs can occur. Both immediate and delayed hypersensitivity to PPIs are uncommon [5, 6]. However, severe, life-threatening immediate reactions, including anaphylaxis, have been reported [7–16]. The real rate of hypersensitive reactions to PPIs is unknown.

The aim of this retrospective study is to evaluate the rate of hypersensitive reactions to PPIs in patients admitted to our Unit of Allergy and Clinical Immunology between 2008 and 2013 due to a history of drug hypersensitivity.

## Methods

The informed consent was collected from patients and we obtained the consent by our local ethical committee (Prot. 26/16, 15-03-2016). We retrospectively analyzed the data of the patients with a history of drug allergy. It were considered eligible patients admitted to the Allergy and Clinical Immunology Division at the University Hospital of Messina between 2008 and 2013. From a database of 1229 patients (921 women, 308 men) with adverse drug reaction (ADR) we extrapolated the data about PPI reactions.

## Results

Twelve patients (10 female, 2 men; mean age 46.5 (± 18.2) out of 1229 patients with ADR (1%) had a positive history for hypersensitive reaction to PPI. Two patients out of 12 were also affected by both allergic rhinitis and food allergy. 9 subjects have had at least another ADR, 5 of them to a NSAID, 3 to an antibiotic, 2 to an anti-hypertensive drug. Obviously, the 12 patients have had a diagnosis of GERD or gastritis; the most frequent co-morbidity other than the above cited were thyroid dysfunction and essential arterial hypertension. Only 5 of them were in taking other drugs (1 patient levothyroxine and an antiplatelet drug, 3 patients anti-hypertensive molecules, and 1 a bisphosphonate drug). Every patient had introduced the PPI as the last molecule in their therapeutic program. On this basis, they re-introduced the other drugs without having ADR apart from the PPI.

**Fig. 1:**
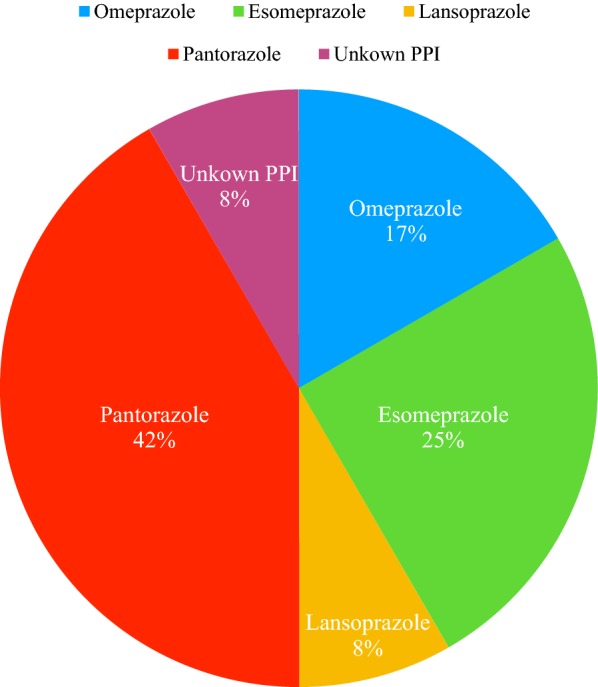
Rate of PPIs ADR

Pantoprazole was the most frequent PPI involved in causing reactions (5 patients), followed by esomeprazole (3 patients), omeprazole (2 patients) and lansoprazole (1 patient); we were unable to identify the exact PPI molecule in one patient (Fig. 1). Table 1 shows the characteristics of patients’ clinical data.

**Table 1:**
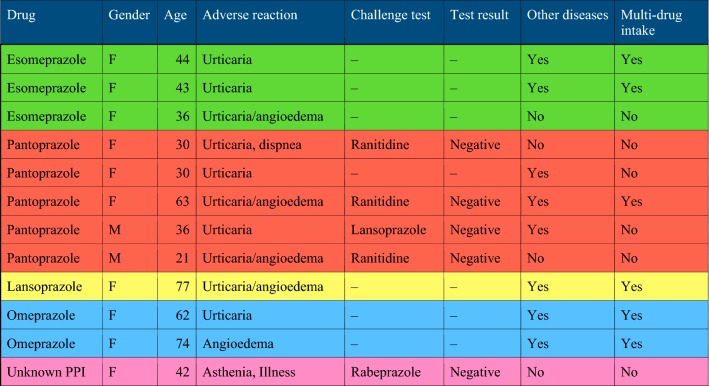
Case series of adverse drug reactions to PPI

**Fig. 2:**
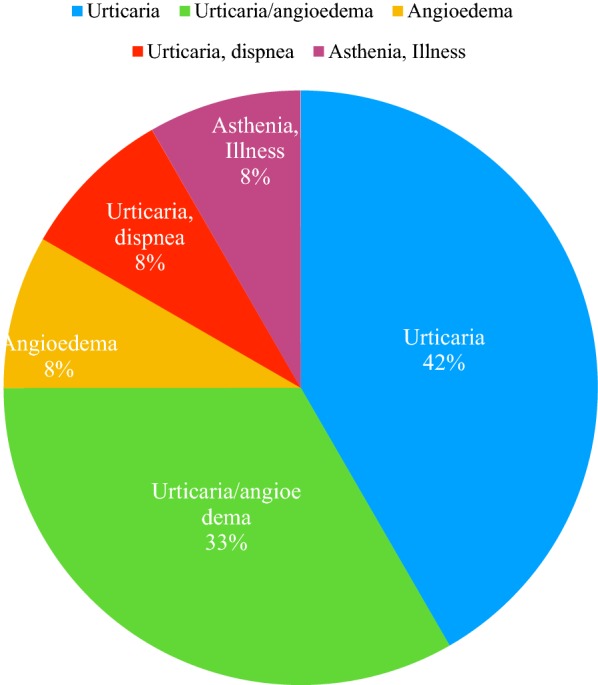
Signs and  symptoms frequency

**Fig. 3:**
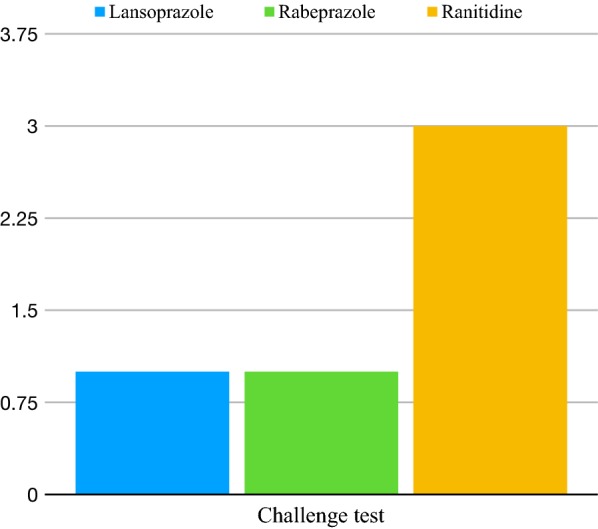
Alternative drugs tested

The most frequent adverse reaction to PPI were urticaria and urticaria/angioedema. Frequency of signs and symptoms are showed in Fig. 2. Based on patient personal history, we performed an oral challenge test for an alternative anti-acid drug on 5 of them. Three of them were challenged for ranitidine, 1 patient was challenged for lansoprazole and the remaining one for rabeprazole (Fig. 3). None of them had adverse reactions, so all the tests were negative.

## Discussion

An increasing number of reports about allergic reactions against PPIs are present in the literature, starting from 1996, as previously reported [17].

By spanning the entire literature, it emerged that every PPI molecule can cause allergic reactions [18–22]. The sensitization rate can differ from a study to the other.

In the Food and Drug Administration Adverse Event Reporting System Public Dashboard (FAERS) the adverse reactions to PPIs represent the 0.37% of the reactions to all the drugs reported from 1989 (when omeprazole was first marketed in the United States) to 2017. These reports include all kinds of adverse events; skin reactions, mainly represented by rash, urticaria, erythema and pruritus, represented the 17% of all adverse events. The cases of anaphylactic shock reported were 131, about the 0.24% of all reactions to PPIs [23].

In 1999, the Uppsala Monitoring Centre database reported 42 cases of anaphylactic reaction to PPIs that constituted the 0.2% of all the adverse reactions to these drugs. The rate of all anaphylaxis cases was the 0.8% of adverse reactions to all drugs (Uppsala). These data agree with FAERS data.

In 2008, in the Italian pharmacovigilance network it were reported 123 adverse events caused by PPIs. 9 of these reactions occurred in our region (Sicily). It occurred 1.41 adverse events for every million of PPI packs used in Italy and 0.86 in Sicily [24]. However, the hypersensitive reactions were very low.

Our study analyzed the incidence of hypersensitive events to PPIs over a 6-year period in a group of patients with a history of drug hypersensitivity. Our data showed a higher rate.

In Literature, the most frequent hypersensitivity reactions rate was associated to omeprazole. These reactions ranged from immediate ones, such as urticaria-angioedema and anaphylaxis, to delayed events, such as allergic contact dermatitis and Drug Rash with Eosinophilia and Systemic Symptoms (DRESS) [5, 15, 25–28].

Also in the FAERS database, omeprazole was the main culprit PPI, followed by lansoprazole [23].

In the Italian pharmacovigilance database, in 2008, the most reported PPIs adverse events were to Lansoprazole. In fact, that was the mainly used PPI molecule in that year [24]. A more recent retrospective study identified Lansoprazole as the most frequent PPI in causing anaphylaxis  [29]

In the present study Pantoprazole was the most common allergic molecule involved. However, in a previous manuscript, we individuated omeprazole as the most frequent PPI in causing hypersensitive reaction, followed by Pantoprazole [17].

## Conclusion

According to our experience and according to literature and pharmacovigilance reports, ADR caused by PPIs are ever increasing. The data about the hypersensitive rate of each molecule can varies depending on the study considered. The differences are probably due to the historical period considered. In fact, there is a higher rate for the older molecules in the less recent studies and an increasing one for new molecules during the last years.

Moreover, it is very difficult to compare the data available because the populations studied are very heterogenic and the cohorts of patients are not comparable. Most studies report only the number of cases of adverse events. On the contrary, the pharmacovigilance database reports the number of cases reported, which are probably underestimated.

Furthermore, spanning Literature we did not find a study similar to our own in order to compare the results.

According to the report of pharmacovigilance, ADR are still under-reported, at least in our region; however, considering the frequency of their prescription worldwide, the risk of severe allergic events is low.

Further studies are needed to provide clearer data on the real incidence and prevalence of hypersensitive reactions to each PPI molecule. Future investigations should be useful to help physician in choosing the best molecule to prescribe and, in case of hypersensitivity, the alternative molecule to test, also considering the possibility of cross-reactions.
